# Surgical Operation

**Published:** 1845-08-01

**Authors:** 


*Surgical operation by Dr. Lewis.*—We perceive by the Boston Journal (July 9th,) that our friend, and quondam anatomical teacher, Winslow Lewis, M. D., of Boston, has recently "amputated the whole arm, embracing the scapula and clavicle, having first taken up the subclavian artery. The operation was rendered necessary in consequence of a terrible accident by machinery. Unfortunately the patient died of the injuries he had received."

